# Pilonidal Disease: A Brazilian epidemiological study with an overview of 45,000 surgeries performed in the Unified Health System

**DOI:** 10.1590/0100-6991e-20260026-en

**Published:** 2026-01-27

**Authors:** FABRICIO DOIN PAZ DE OLIVEIRA, RICARDO DIAS MONT’ALVERNE, SONIA CRISTINA CORDERO TIME, ANA CAROLINA BUFFARA BLITZKOW

**Affiliations:** 1 - Academia do Laser - Concórdia - SC - Brasil; 2 - Center for Inflammatory Bowel Diseases and Coloproctology, Sao Camilo Hospital - Ponta Grossa - PR - Brasil; 3 - Santa Casa de Misericórdia de Fortaleza - Fortaleza - CE - Brasil; 4 - Universidade Federal do Ceará, Hospital Geral Dr Waldemar de Alcântara - Fortaleza - CE - Brasil; 5 - Universidade Federal do Paraná, Hospital de Clínicas - Unidade de Cirurgia geral - Curitiba - PR - Brasil; 6 - Vita Batel Hospital, Departamento de coloproctologia, Curitiba - PR - Brasil

**Keywords:** Pilonidal Sinus, Epidemiology, Surgery, Public Health, Sacrococcygeal Region, Cirurgia Colorretal, Cirurgia Geral, Epidemiologia, Seio Pilonidal, Pesquisa Sobre Serviços de Saúde

## Abstract

**Introduction:**

Pilonidal disease (PD) is a chronic inflammatory disorder affecting the skin and subcutaneous tissues of the sacrococcygeal region, with a predominant impact on young adults. Although there have been considerable advances in surgical management, national-level epidemiological data on PD in Brazil remain scarce.

**Methods:**

We conducted a retrospective, descriptive, population-based study analyzing all surgical procedures for PD recorded in Brazil’s public health system (SUS) from January 2014 to December 2023. Data were extracted from the DATASUS platform, using specific procedural codes for pilonidal sinus. Prevalence rates per 100,000 inhabitants were calculated utilizing population estimates from the Brazilian Institute of Geography and Statistics (IBGE). Temporal trends were assessed through linear regression analysis, and regional comparisons were performed using Pearson’s chi-square test with Bonferroni correction.

**Results:**

Throughout the ten-year period, 45,915 patients underwent surgery for PD, yielding a mean prevalence of 2.42 cases per 100,000 inhabitants. The highest regional prevalence was observed in the South, while the North had the lowest rates. An overall upward trend in surgical intervention rates was noted, particularly from 2014 to 2019, with a temporary decline during the COVID-19 pandemic (2020-2021) and subsequent recovery. Significant regional disparities were evident, suggesting that healthcare infrastructure and access differences may contribute to these patterns.

**Conclusion:**

Pilonidal sinus disease (PSD) has shown a steady rise in surgical cases in Brazil, particularly in the South and Southeast regions. Despite lower national prevalence compared to high-income countries, the increasing trend highlights growing public health concerns and significant regional disparities.

## RESUMO

**Introdução:**

A doença pilonidal (DP) é uma condição inflamatória crônica que acomete a pele e o tecido subcutâneo da região sacrococcígea, afetando predominantemente adultos jovens. Apesar dos avanços consideráveis na abordagem cirúrgica, os dados epidemiológicos nacionais sobre DP no Brasil ainda são escassos.

**Métodos:**

Foi realizado um estudo retrospectivo e descritivo incluindo todos os procedimentos cirúrgicos para DP registrados no Brasil, no SUS, entre janeiro de 2014 e dezembro de 2023. Os dados foram extraídos da plataforma DATASUS, utilizando códigos de procedimento específicos. As taxas de prevalência por 100.000 habitantes foram calculadas com base em estimativas populacionais fornecidas pelo IBGE. As tendências temporais foram avaliadas por análise de regressão linear, e as comparações regionais foram realizadas utilizando o teste do qui-quadrado de Pearson.

**Resultados:**

Durante o período de dez anos, 45.915 pacientes foram submetidos à cirurgia para DP, com uma prevalência média de 2,42 casos por 100.000 habitantes. A maior prevalência foi observada no Sul, enquanto o Norte apresentou as menores taxas. Observou-se uma tendência geral de aumento nas taxas de intervenções cirúrgicas, especialmente entre 2014 e 2019, com um declínio temporário durante a pandemia de COVID-19 e recuperação subsequente. Disparidades regionais significativas foram identificadas.

**Conclusão:**

A doença pilonidal (DP) vem apresentando um crescimento contínuo no número de cirurgias no Brasil, com destaque para as regiões Sul e Sudeste. Embora a prevalência nacional ainda seja inferior à observada em países de alta renda, a trajetória ascendente revela não apenas um problema emergente de saúde pública, mas também desigualdades regionais significativas..

## INTRODUCTION

Pilonidal cyst, also known as sacrococcygeal cyst or pilonidal disease (PD), is a chronic inflammatory condition that affects the skin and subcutaneous tissue, occurring predominantly in the sacrococcygeal region. The first description of this disease dates back to the 1830s, by British surgeon Herbert Mayo^1^. Despite decades of study, defining the exact mechanisms involved in the development of pilonidal disease remains a challenge. Nevertheless, several contributing factors have been consistently reported, including overweight and obesity, sedentary lifestyle, history of local trauma, presence of deep intergluteal cleft, high hair density in the region, poor hygiene, family history, and conditions such as polycystic ovary syndrome^2^ ^,^ ^3^.

In recent years, surgical treatment of pilonidal disease has evolved significantly. Innovative and minimally invasive techniques, such as endoscopic pilonidal sinus treatment (EPSiT) using a fistuloscope, and laser ablation therapies, such as Sinus Laser Therapy (SILAT) and Minimum Energy Laser Pilonidotomy (MELPi), have shown promising results compared to traditional approaches involving wide excision, suturing, and curettage^4^ ^-^ ^6^. These advances seek to accelerate patient recovery, reduce recurrence rates, and minimise postoperative morbidity.

Despite advances in therapeutic options, epidemiological data on PD in Brazil are still limited. While studies in the United States of America (USA) report a prevalence of approximately 26 cases per 100,000 inhabitants^7^, European data suggest an incidence of about 3.1 cases in men for every case in women per 100,000 individuals8. Given this gap in national data, the present study aims to describe the incidence patterns and epidemiological characteristics of patients undergoing surgical treatment for PD within the Unified Health System (Sistema Único de Sáude - SUS).

## METHODS

This study evaluated all patients who underwent surgery for PD in the Brazilian Unified Health System (SUS) over a ten-year period, between January 2014 and December 2023. It was designed as a retrospective, descriptive, population-based study, using exclusively publicly available data.

The information was extracted from DATASUS, the official platform of the Ministry of Health that collects records of hospital admissions throughout the country. To ensure that only cases of surgery for pilonidal disease were considered, specific procedure codes referring to pilonidal sinus were used.

As this study used public, anonymised and freely accessible data, there was no need for approval by a Research Ethics Committee, in accordance with Resolution No. 510/2016 of the National Health Council.

Prevalence was calculated based on the ratio between the number of surgeries recorded and the population estimated by the Brazilian Institute of Geography and Statistics (Instituto Brasileiro de Geografia e Estatística - IBGE) for each year of the period analysed. The results were expressed as the number of procedures per 100,000 inhabitants, which allowed comparisons between different regions and over the time series.

In addition to descriptive analysis, the trends were investigated over time using linear regression models, which enabled us to assess whether there was an increase or decrease in surgeries and to what extent. Comparisons between the five regions of Brazil were performed using Pearson’s chi-square test, with Bonferroni Correction, in order to verify whether the differences observed were consistent or could be attributed to chance. All statistical analyses were performed using JMP Pro 13 software (SAS Institute Inc., Cary, NC, USA).

## RESULTS

**Figure 1:**
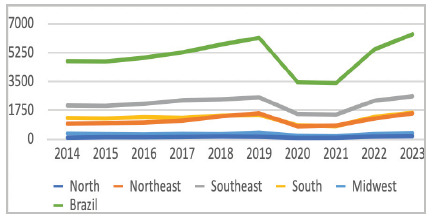
Evolution of the absolute number of cases over time by region.

Between 2014 and 2023, 45,915 surgeries were performed to treat PD in Brazil. This corresponds to an annual average of 4,591 procedures, with values ranging from 3,405 in 2014 to 6,366 in 2023. After adjusting for population, the national average rate was 2.42 cases per 100,000 inhabitants, ranging from 1.60 to 3.13 cases per 100,000 in the period analysed (Table 1, Figure 1).

**Table 1:** Rates of surgical procedures per 100,000 inhabitants by region (2014-2023).

Year	North	Northeast	Southeast	South	Central-West	Brasil
2014	0.55	1.68	2.40	4.46	2.32	2.33
2015	0.70	1.73	2.36	4.32	2.09	2.31
2016	0.68	1.78	2.50	4.58	1.93	2.40
2017	0.76	1.96	2.71	4.41	2.13	2.54
2018	0.94	2.45	2.76	4.83	1.98	2.75
Year	North	Northeast	Southeast	South	Central-West	Brasil
2019	0.82	2.76	2.88	4.91	2.49	2.92
2020	0.44	1.37	1.73	2.82	1.31	1.64
2021	0.48	1.44	1.67	2.61	1.17	1.60
2022	0.92	2.27	2.67	4.44	1.97	2.62
2023	1.10	2.88	3.07	5.38	2.35	3.13

The years with the lowest prevalence were 2020 and 2021, coinciding with the peak of the Coronavirus Disease-2019 (COVID-19) pandemic, when rates fell to 1.60 and 1.64 cases per 100,000 inhabitants. In contrast, 2023 recorded the highest prevalence in the series, with 3.13 cases per 100,000 inhabitants.

**Figure 2:**
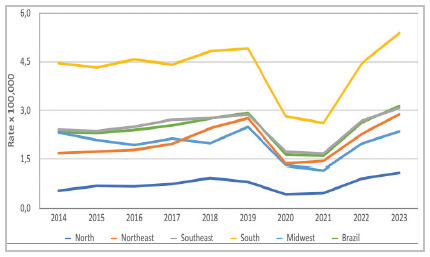
Annual prevalence rates (per 100,000) in the five geographical regions of Brazil.

In the regional analysis, significant disparities were observed. The South consistently had the highest rates, with an average of 4.27 per 100,000 inhabitants, ranging from 2.61 to 5.38. The North had the lowest rates, with an average of 0.739 per 100,000 inhabitants (0.44 to 1.10). The Northeast, Southeast, and Central-West regions showed intermediate values that were relatively close to each other, with averages of 1.82 (1.37-2.88), 2.47 (1.67-3.07), and 1.97 (1.17-2.49), respectively (Figure 2).

At the national level, there was a trend of continuous growth in surgeries from 2014 to 2019, interrupted during the pandemic years (2020-2021) and resumed in 2022 and 2023. Excluding the pandemic years from the trend analysis, the annual rate of increase was 0.08 points per year (95% CI: 0.02-0.13; p = 0.0012).

**Figure 3:**
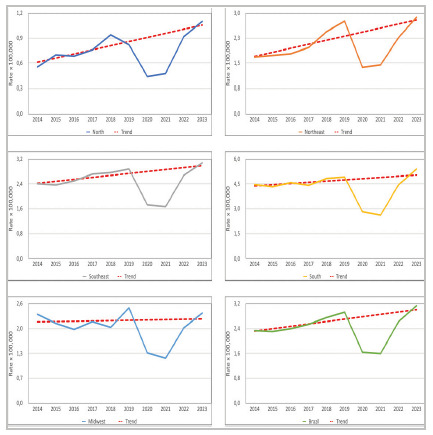
National trend line for prevalence rates excluding 2020-2021 (years impacted by COVID-19).

**Figure 4:**
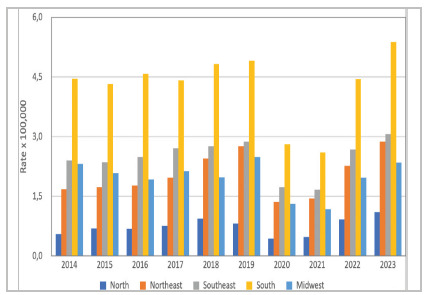
Analyses of linear trends specific to the region.

In the regional analysis, the Northeast showed the most pronounced growth, with β = 0.12/year (95% CI: 0.04-0.20), followed by the North, with β = 0.05/year (95% CI: 0.03-0.07), and the Southeast, with β = 0.06/year (95% CI: 0.02-0.10). The South showed a weaker trend, β = 0.07/year (95% CI: -0.01-0.16), without reaching statistical significance (p = 0.07). The Central-West did not show a significant trend, β = 0.01/year (95% CI: -0.06-0.07; p = 0.756), suggesting stability over the period (Table 2, Figures 3 and 4).

**Table 2:** Comparative analysis of annual growth trends by region (linear regression coefficients).

Region	Annual growth (β)	CI 95%	p-value
Brazil	0.08	0.02-0.13	0.0012
North	0.05	0.03-0.07	<0,01
Northeast	0.12	0.04-0.20	<0,01
Southeast	0.06	0.02-0.10	<0,01
South	0.07	-0.01-0.16	0.07
Central-West	0.01	-0.06-0.07	0.756

Statistical comparisons between regional prevalences confirmed that the South had significantly higher rates than all other regions (p < 0.001). On the other hand, the North had significantly lower rates in all years (p < 0.001). Some overlaps in rates occurred between the Northeast and the Central-West (no significant difference in 2016, 2017, and 2019-2022) and between the Southeast and the Central-West (no difference in 2014, 2015, and 2019). The Northeast and Southeast also had similar values in 2019 and 2023.

These results confirm a growing trend in surgical interventions for PD in Brazil, especially in regions with better health infrastructure, while emphasising disparities that warrant further investigation and policy action.

## DISCUSSION

This study represents the first comprehensive, nationwide epidemiological analysis of surgically treated cases of PD in Brazil, based exclusively on data from the Unified Health System (SUS).

Our findings demonstrate a progressive increase in surgical interventions for PD throughout the country. Interestingly, similar upward trends have been documented internationally. For example, Oetzmann et al.8 reported a 33% increase in hospitalisations for PD over a 13-year period in Germany, accompanied by substantial regional variability. In Brazil, although overall prevalence rates remain lower, an upward trend was evident, with 2023 recording the highest prevalence in the study period. These findings suggest that broader factors, including changes in lifestyle behaviours, increased sedentary habits, and improvements in healthcare access, may be contributing to the overall increase in the cases of PD.

Both our data and the German study by Oetzmann et al.^8^ reveal pronounced regional disparities in the incidence of PD. In Germany, differences between federal states were notable, although specific causal factors have not been definitively identified. Similarly, in Brazil, the South consistently had the highest surgical prevalence, while the North had the lowest rates. These observations support the hypothesis that multifactorial determinants-including variations in health infrastructure, socioeconomic conditions, demographic characteristics, and local medical practices-may generate regional differences^8^ ^,^ ^9^.

Specifically, the high rates of surgery observed in southern Brazil likely reflect better access to healthcare, more organised referral systems, and a higher concentration of specialised surgeons. This aligns with previous findings indicating that the quality of healthcare infrastructure is a critical determinant of variations in surgical prevalence^10^. Furthermore, previous studies suggest that demographic factors, environmental exposures, and access to surgical specialists may further influence these regional discrepancies^8^ ^,^ ^9^.

There is a notable lack of studies assessing the true incidence of PD. Sondenaa et al.^7^, in a population-based study conducted in Norway, reported an incidence of 26 cases per 100,000 inhabitants. However, given that this estimate dates back more than two decades, its applicability to current epidemiological patterns is limited. More recent investigations continue to show an increasing incidence of PD in developed countries, although the precise reasons behind this trend remain unclear^8^ ^-^ ^12^.

The COVID-19 pandemic had a significant impact on the trajectory of surgical procedures for PD. We observed a decline in surgeries during 2020 and 2021, consistent with global reports attributing reductions in elective procedures to the reorganisation of the healthcare system, prioritisation of emergency care, and reduced patient care during periods of high viral transmission^13^. In Brazil, the overload of hospital resources during the pandemic likely exacerbated the decline, with an evident recovery in 2022-2023 as services resumed and postponed cases were resolved.

Another important epidemiological feature of PD is its well-documented male predominance, with reported male-to-female ratios ranging from 2:1 to 3:1^8^ ^,^ ^14^. This disparity is believed to be related to factors such as androgen-mediated hair growth, deeper natal clefts, and specific occupational exposures. Unfortunately, our database did not allow for stratification by sex, but future studies focusing on specific gender differences are needed.

The PD is most commonly diagnosed among young men, with a mean age at diagnosis of approximately 30 years. It is estimated to occur approximately four times more frequently in men than in women^15^. Given its typical onset during the peak of educational and professional productivity, PD has substantial public health and socioeconomic implications, including impacts on quality of life and absenteeism.

Occupational risk factors have long been recognised in the epidemiology of PD. The term “jeep disease” was coined during World War II to describe the high incidence of PD among military personnel exposed to prolonged sitting and vibration. Contemporary studies have confirmed the increased risk among individuals engaged in sedentary occupations, such as drivers and office workers^3^ ^,^ ^16^, a finding that may partially explain higher rates in more urbanised and service-oriented regions.

Ethnicity also appears to play a role in the distribution of PD. Research suggests that PD is more prevalent among individuals of Caucasian descent, while lower incidence rates are observed among African and Asian populations^15^ ^,^ ^17^. Such phenotypic differences may help explain some of the regional statistical disparities identified in Brazil.

Recognised risk factors for PD include family history of the disease, follicular bacterial colonisation, mechanical trauma to the skin, excessive hairiness (especially coarse hair), hyperhidrosis, obesity, sedentary lifestyle, and inadequate ventilation of the gluteal cleft area^2^ ^,^ ^7^ ^,^ ^15^ ^,^ ^18^. The continuous increase in the prevalence of PD may therefore reflect the progressive amplification of these lifestyle-related factors in modern societies.

Although PD is generally considered a benign condition, its untreated or recurrent forms can lead to substantial morbidity. Chronic cases may involve multiple sinus tracts, abscess formation, and persistent impairment of quality of life. In rare cases, chronic inflammation may progress to malignant transformation, most commonly to squamous cell carcinoma^19^ ^-^ ^21^, emphasising the critical need for timely diagnosis and effective treatment.

Surgical intervention remains the cornerstone of PD treatment. Traditional techniques, such as wide excision with secondary healing or flap reconstruction, continue to be widely used. However, in recent years, we have seen the emergence of minimally invasive approaches, including EPSiT^4^ and laser-based therapies such as Sinus Laser Ablation of the Cystic Duct (SILAC), SILAT, and MELPi^6^ ^,^ ^22^ ^-^ ^24^. These new techniques have demonstrated comparable efficacy, with lower recurrence rates, reduced postoperative pain, and a faster return to daily activities, although a slightly higher treatment failure rate (~10%) has been observed^25^.

Despite promising results, access to minimally invasive procedures remains largely restricted to high-resource settings and private healthcare institutions in Brazil. Expanding availability within the public healthcare system represents a critical future priority.

The economic burden of PD is also noteworthy. In the United Kingdom (UK), pilonidal disease has been associated with substantial healthcare costs and productivity losses related to prolonged recovery periods and recurrent disease^26^. In Brazil, although national-level cost analyses are lacking, the growing volume of surgical procedures suggests a growing financial impact on the public healthcare system. Cost-effectiveness studies comparing conventional and minimally invasive techniques are urgently needed to guide the efficient allocation of resources.

This study has certain limitations. Reliance on administrative databases restricted the analysis to cases treated surgically in public hospitals, excluding patients treated conservatively, those treated in private healthcare facilities, and potentially misdiagnosed cases. In addition, detailed clinical data, such as disease severity, surgical technique used, and recurrence rates, were not available. Despite these limitations, the strength of the study lies in its large sample size, standardised national data, and longitudinal design, which provide valuable information on the epidemiology of PD in Brazil.

Future research should include national registries that integrate the public and private sectors, with detailed clinical data and follow-up on recurrences. At the same time, health policies need to prioritise prevention, early diagnosis, and greater access to modern surgical techniques in order to reduce the burden of PD and make care more equitable across the country.

## CONCLUSION

The PD has shown continuous growth in the number of surgeries performed in Brazil, with a higher concentration in the South and Southeast regions. Although the national prevalence is still lower than that observed in high-income countries, the upward trend highlights an emerging public health problem and reinforces the existence of regional inequalities.
